# ‘Real time’ monitoring of antipsychotic prescribing in patients with dementia: a study using the Clinical Record Interactive Search (CRIS) platform to enhance safer prescribing

**DOI:** 10.1136/bmjoq-2019-000778

**Published:** 2020-03-29

**Authors:** Craig Colling, Christoph Mueller, Gayan Perera, Nicola Funnell, Justin Sauer, Daniel Harwood, Robert Stewart, Delia Bishara

**Affiliations:** 1 Biomedical Research Centre (BRC), Institute of Psychiatry, Psychology and Neuroscience, London, UK; 2 Mental Health of Older Adults and Dementia Clinical Academic Group (SLaM), South London and Maudsley NHS Foundation Trust, London, UK

**Keywords:** dementia, checklists, clinical audit, continuous quality improvement, electronic health records

## Abstract

**Background:**

The use of antipsychotic drugs in dementia has been reported to be associated with increased risk of cerebrovascular events and mortality. There is an international drive to reduce the use of these agents in patients with dementia and to improve the safety of prescribing and monitoring in this area.

**Objectives:**

The aim of this project was to use enhanced automated regular feedback of information from electronic health records to improve the quality of antipsychotic prescribing and monitoring in people with dementia.

**Methods:**

The South London and Maudsley NHS Foundation Trust (SLaM) incorporated antipsychotic monitoring forms into its electronic health records. The SLaM Clinical Record Interactive Search (CRIS) platform provides researcher access to de-identified health records, and natural language processing is used in CRIS to derive structured data from unstructured free text, including recorded diagnoses and medication. Algorithms were thus developed to ascertain patients with dementia receiving antipsychotic treatment and to determine whether monitoring forms had been completed. We used two improvement plan-do-study-act cycles to improve the accuracy of the algorithm for automated evaluation and provided monthly feedback on team performance.

**Results:**

A steady increase in antipsychotic monitoring form completion was observed across the study period. The percentage of our sample with a completed antipsychotic monitoring form more than doubled from October 2017 (22%) to January 2019 (58%).

**Conclusion:**

‘Real time’ monitoring and regular feedback to teams offer a time-effective approach, complementary to standard audit methods, to enhance the safer prescribing of high risk drugs.

## Introduction

Behavioural and psychological symptoms of dementia include agitation, aggression, hallucinations, delusions and disinhibition.^1^ These symptoms are common in dementia, can be very distressing to both patients and carers, may require medication and are often difficult to treat. For many years, antipsychotic drugs had been widely used to manage these neuropsychiatric symptoms; however, in 2004, their use in dementia came under scrutiny following reports of possible increased risk of cerebrovascular events (CVEs) and mortality.^2 3^ Concerns arose from reviews of both published and unpublished clinical trials and led to manufacturers and health regulatory agencies issuing warnings worldwide. The UK Department of Health subsequently commissioned the ‘Time for Action’ report^4^ which raised awareness of the risks of these drugs for people with dementia. Since then, there has been a national drive in the UK to reduce the use of antipsychotic drugs in patients with dementia and improve the safety of prescribing of these drugs. Similar campaigns were carried out in other parts of Europe,^5^ the USA^6^ and Australia.^7^

The Prescribing Observatory for Mental Health (POMH-UK)^8^ is a subscription-based project established in 2005 by the Royal College of Psychiatrists that helps specialist mental health services across the UK improve the safety and quality of their prescribing practice. Services that are members of POMH-UK take part in audit-based quality improvement programmes (QIPs), which focus on specific topics within mental health prescribing. The prevalence and quality of antipsychotic drug prescribing in people with dementia have been a regular audit on the POMH-UK agenda, and data for this have been collected in 2011, 2012 and 2016. The standards for this audit are derived from the National Institute for Health and Care Excellence (NICE) Guideline for Dementia.^1^

Increasing digitalisation of health records has created novel opportunities for automated feedback of information at scale, used to drive improvements in clinical practice. The purpose of the project described here was to evaluate such an initiative to enhance the quality of antipsychotic prescribing and monitoring for people with dementia by providing automated, regular feedback on team performance.

## Methods

### Setting

The South London and Maudsley NHS Foundation Trust (SLaM) is one of Europe’s largest healthcare providers for mental health and dementia and serves a local population of 1.25 million residents.^9^ SLaM is a member of POMH-UK and has participated in all relevant QIPs over the years. While the number of people with dementia when prescribed an antipsychotic drug has remained relatively low in SLaM, our Trust’s POMH-UK audit report had shown that the performance on certain quality of prescribing standards had declined over the years (data available on request). As a result, SLaM introduced ‘antipsychotic in dementia’ monitoring forms incorporated into its electronic health records. Two forms were designed: the first included all the relevant considerations and actions to be taken before starting a patient with a diagnosis of dementia on an antipsychotic drug (see online supplementary appendix 1). The second form was designed to document the clinical review of the person taking the drug; this has a checklist of side effects and physical health monitoring requirements, and space to record risk benefit analysis and the outcome of the review (see online supplementary appendix 2). It is recommended that an antipsychotic review form is completed every 2–4 weeks for in-patients and every 3–6 months for community patients; however, not all sections need to be completed each time. A copy of the forms is available in the supplementary information (see online supplementary appendix 1 and 2).

10.1136/bmjoq-2019-000778.supp1 Supplementary data

The electronic antipsychotic monitoring forms were designed to include all the relevant POMH-UK prescribing and monitoring standards as set out in their antipsychotic in dementia audit tool. The forms were piloted by in-patient and community consultants for a year and then advice sought on potential improvements to be made. A more user-friendly version was consequently developed and re-incorporated into the electronic health records. Implementation of the use of these monitoring forms included dissemination of SLaM’s POMH-UK audit results (showing the need for improvement on certain prescribing standards), with advice on using the forms and their location in the electronic health records. This was presented at SLaM’s drug and therapeutic and quality governance committees. In addition, this information was also included in the Trust’s Medicines Bulletin (which is circulated to all prescribers), and incorporated into junior doctors’, nurses’ and pharmacists’ medication training programmes.

### Patient and public involvement

Patients were not involved in this process.

### Intervention

Data for this study were obtained from SLaM’s Clinical Record Interactive Search (CRIS) platform. This provides research access to more than 400 000 mental health records within a robust governance framework.^9^ CRIS development has included natural language processing (NLP) techniques to generate structured metadata from unstructured free text^9^; these include data on recorded diagnosis and medication,^10 11^ substantially supplementing data on these entities from structured fields. In the initiative described here, we combined NLP-derived data with data from structured fields to identify patients with a recorded dementia diagnosis and recorded antipsychotic agent receipt. We combined this with an automated extraction of structured data from antipsychotic monitoring forms to ascertain their completion or not at given census points.

**Figure 1:**
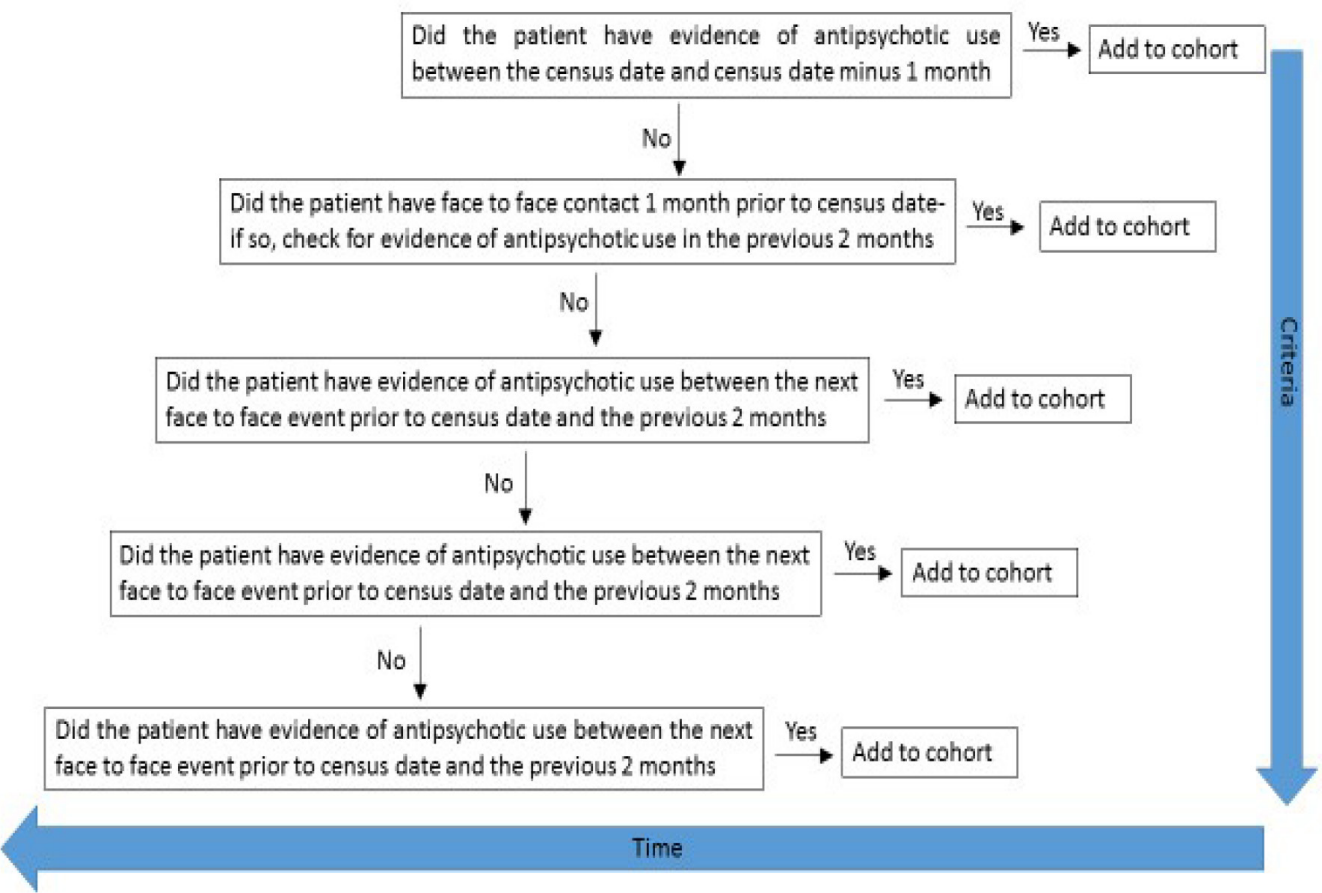
Criteria used to identify recent antipsychotic use.

Regarding cohort definition, using CRIS we selected patients receiving active care from mental health of older adults (MHOA) community mental health teams (CMHTs), MHOA care home intervention teams (CHITs) or MHOA inpatient services at a defined census date (28th of each month) who had a dementia diagnosis (defined as F00-F03 in Internation Classification of Diseases, Tenth Revision (ICD-10) fields or relevant diagnostic terms ascertained through NLP) prior to the census date and evidence of recent antipsychotic use. Figure 1 summarises the algorithm used to identify recent antipsychotic use.

We also generated secondary output in which we excluded patients who had ever received a psychosis diagnosis (ICD-10 codes F20–F29), or those who received a different psychiatric diagnosis after the index dementia diagnosis. This output was generated following clinician feedback in order to exclude patients with dementia and a comorbid psychotic disorder who may need to be treated with an antipsychotic drug for this indication, as well as excluding people whose original dementia diagnosis might have been superseded by that of another mental disorder.

### Strategy

The antipsychotic monitoring form was first introduced into SLaM in 2015 (see online supplementary appendix 1 and 2). We introduced a monthly team level monitoring report in October 2017 to feed back aggregate team performance to consultant psychiatrists and team leaders. Team performance consisted of the percentage of form completion for each team. This is based on the number of forms that should have been completed, as predicted by the algorithm identifying the number of patients with dementia currently prescribed an antipsychotic drug. In addition, to the monthly aggregate monitoring report we asked consultant psychiatrists to consider reviewing their team caseloads with de-anonymised data from the algorithm output to identify instances where the algorithm might be refined. We used two improvement plan-do-study-act (PDSA)^12^ cycles to improve the reliability of the algorithm. We collected our outcome data continuously throughout to test the results of each improvement cycle.

#### PDSA cycle 1

The recommendation from clinical services in January 2018 was to identify, and feed back, the number of antipsychotic monitoring forms completed for cases of dementia where antipsychotic use had not been identified by the algorithm, as it was envisaged that the algorithm might miss some instances and underestimate team performance on monitoring.

#### PDSA cycle 2

The feedback from the clinical services in July 2018 related to increasing the sensitivity of the algorithm for *recent* antipsychotic use by updating the recent antipsychotic use filter to: (1) exclude patients with only one antipsychotic drug reference within the window; and (2) exclude medications with at least one ‘stop’ reference within the recent window. Second, the criterion for additional output was amended to also exclude episodes of patients who had received a different psychiatric diagnosis after their index diagnosis of dementia, as described earlier.

## Results

**Figure 2:**
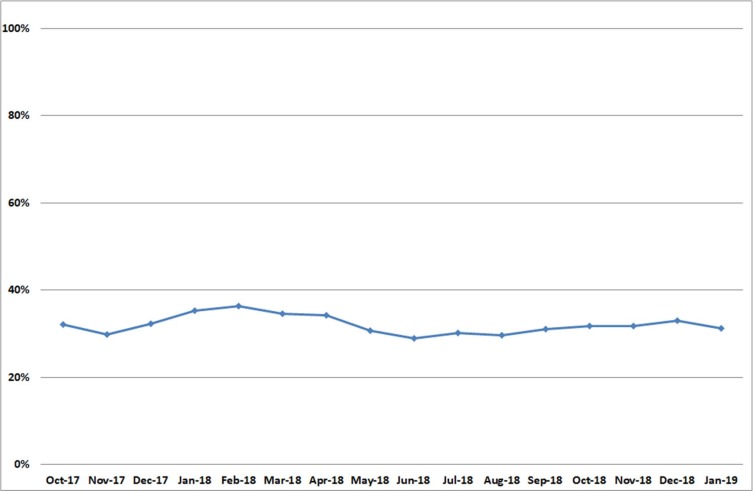
Percentage of patients with dementia with recorded antipsychotic use.

From October 2017 to January 2019, the average monthly number of patients with dementia receiving antipsychotic treatment was 212, representing 32.1% of the dementia caseload; this proportion ranged from 28.9% to 36.6% between the census points, but there was no marked temporal trend (figure 2).

**Figure 3:**
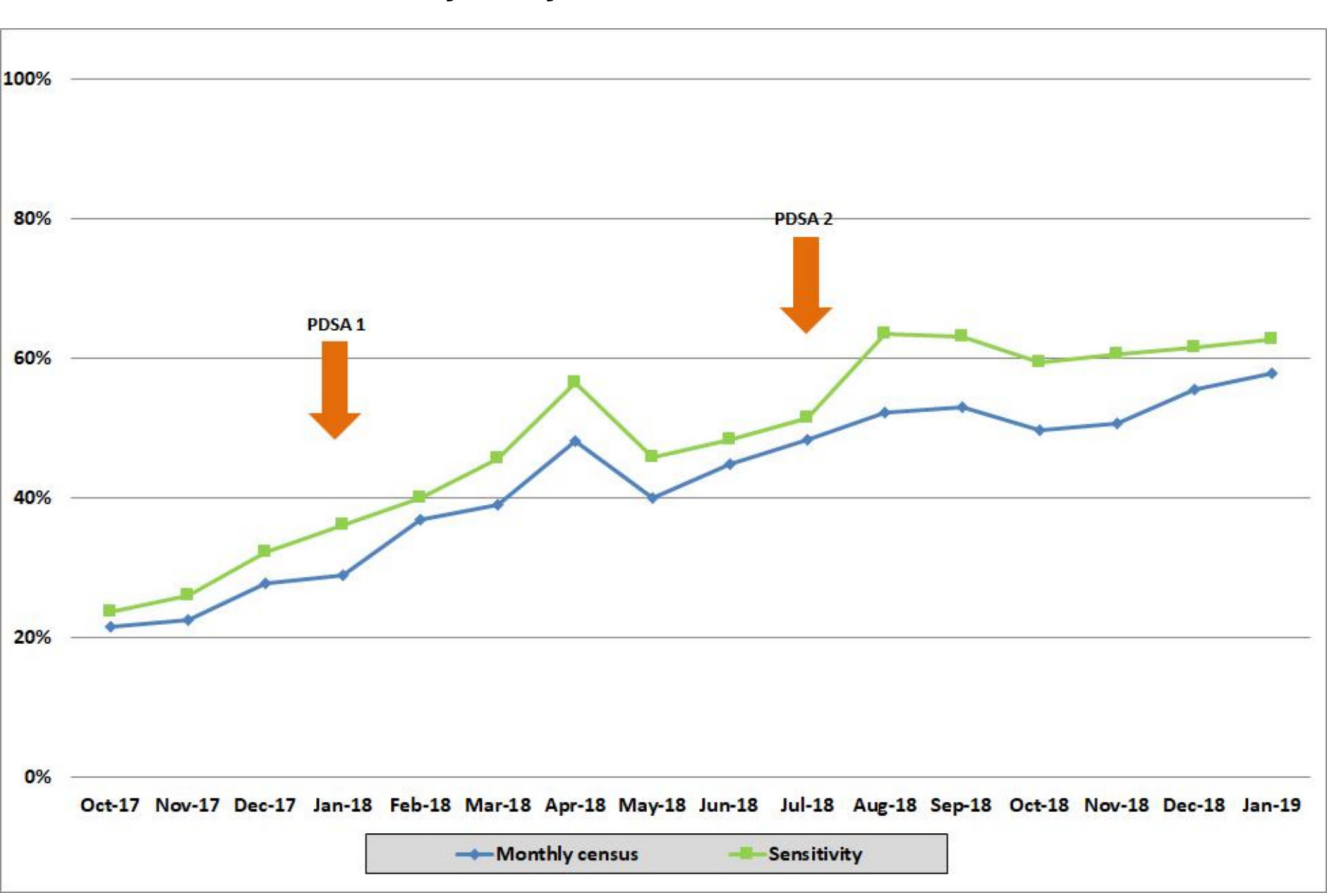
Percentage of antipsychotic monitoring forms completed for monthly census and after sensitivity analysis.

The percentage of our sample with a completed antipsychotic monitoring form more than doubled from October 2017 (21.6%) to January 2019 (58.0%). Figure 3 shows a steady increase in monitoring form completion over this period, based on our main outcome which is represented by the blue line. The sensitivity analysis in October 2017 highlighted that the total people with dementia at that census was 638, of whom 122 (19.0%) had a previous psychosis diagnosis. The antipsychotic monitoring form completion rate after applying the sensitivity analysis was 23.8%, as represented by the green line.

### PDSA cycle 1

In January 2018, there were 462 current patients with a dementia diagnosis without algorithm-estimated recent antipsychotic use, 18 (3.9%) of whom had an antipsychotic monitoring form completed.

### PDSA cycle 2

In July 2018 we updated the algorithm to increase its sensitivity for identifying antipsychotic use, as described. The baseline performance for antipsychotic monitoring form completion was 44.9% (119/265) which rose to 48.5% (96/198) following exclusion of those with only a single mention of an antipsychotic or a stopping reference, and then rose to 60.2% (74/123) following further exclusion of patients who had received a different mental disorder diagnosis after the index dementia diagnosis.

## Discussion

The digitalisation of health records has created an unprecedented volume of information derived from routine care with high potential to transform the way in which services are monitored and tailored to the individual. In particular, programming capability and computer capacity are likely now to be at a sufficient stage of development to support the generation of algorithms to inform clinicians and enable service decision-making based on real-time ‘big data’ derived from electronic health records. However, this depends on the availability, accuracy and quality of information recorded, as well as on achieving pipelines and platforms for information processing and delivery. In mental healthcare, a key challenge has been the fact that most clinically valuable information is recorded in text fields within the health record, such as case notes and correspondence, rather than as structured data points. One approach is therefore to derive data from these text fields using NLP and thus enhance the structure of the record and information available. This has been demonstrated to be achievable in the mental health record through a range of research applications, particularly using the CRIS data resource at SLaM^13–19^ and creates opportunities for algorithms to improve clinical quality such as enhancing prescribing practice as described here.

Digital health records create at least the flexibility of information repositories within which models can be developed and optimised without the need for repeated data collection, as well as the potential for independent validation—across time periods, as adopted in our analyses, or between service providers. In this case, we used a simple form or checklist in combination with the CRIS platform, enhanced through NLP, to achieve efficient and up to date data collection.

Antipsychotic drugs are associated with increased risk of CVEs and mortality in people with dementia.^3^ Despite worldwide campaigns to reduce their use in this patient group, some patients will still require treatment with these drugs, as a last resort.^4^ Hence, enhancing the safety of prescribing and ensuring adequate and regular monitoring of these agents becomes vital, and an impetus for the antipsychotic monitoring initiative was that SLaM had shown inconsistent performance on certain prescribing standards over the years.

A steady increase in antipsychotic monitoring form completion was observed across the study period, and the monitoring and feedback will continue now that the process has been established. The main purpose of the PDSA cycles was to improve the accuracy of the algorithm to detect patients with dementia receiving antipsychotic treatment and levels of form completion. The PDSA cycles were not intended to directly increase completion of the form; however, by improving algorithm accuracy, they may have improved clinician confidence in the algorithm and thereby indirectly improved form completion. In addition, the continuous electronic monitoring of the forms and direct communication with clinicians are at least one way to improve engagement and encourage accurate monitoring. Here, we have displayed the effect of emailing grouped-level patient data to teams and are now also starting to email individual-level patient data as a precursor to creating a real-time dashboard. We believe a real-time dashboard could potentially help with the ‘ceiling effect’ of form completion that we have seen, but this remains to be evaluated.

The aspiration is that this, in turn, will reduce the rate and enhance the quality of prescribing and result in longer-term health benefits; however, this requires longer follow-up and further evaluation of this initiative, as well as potentially its evaluation through multi-site randomisation. Our view is that an improvement from 20% to 60% completion is a highly positive achievement over 15 months of implementation, although clearly further improvement remains an aspiration. Of relevance, the monitoring algorithm was relatively straightforward to set up, once supporting data resources were available, and virtually cost-free in continued implementation. We also plan to investigate the quality of completion of the forms and to identify areas which could be improved. For example, we may find that certain blood tests or an ECG are not being carried out in some teams prior to commencing the antipsychotic drug, or that discussions with relatives are not being documented on the forms. The overall aim is to identify ways to improve the quality and safety of patient care and to standardise this across the trust in a continuous and sustainable way.

Strengths of this initiative and its evaluation include the large samples, naturalistic setting and the ability of CRIS to provide both ‘real-world’ and ‘real-time’ information on routine mental healthcare. In addition, the incorporation of NLP algorithms enabled data sources to be both large and deep as previously described.^20^ One of the key advantages to our approach, considering potential future applicability, is that there was no additional ‘data entry’ required by clinical staff; however, the project did allow teams to view individualised data in order to feed back valuable information for algorithm optimisation. This was allowed through the use of a pseudonymised rather than irreversibly anonymised data resource, enabling provision of identifiable data to teams under evaluation without contravening information governance (since the data remained within the source firewall, were transferred securely and only made available to team members who would have had access to source records for those patients). Feedback loops for algorithm optimisation predominantly involved email communications with developers; however, future initiatives of this nature could readily incorporate more automated learning mechanisms.

Considering potential limitations, this study was based at a single site and at least some findings might reflect local issues and service provision. However, in this respect, efforts were made to ensure the design decisions were potentially generalisable. The NLP-derived data presented here were obtained using relatively simple techniques; these provided increased depth to the record-derived data that could be enhanced further as technology improves. The data sources within SLaM’s health record are relatively heterogeneous, rendering them potentially applicable to a wide range of clinical services/specialities, Finally, clearly this is an observational study and it cannot be concluded that the improvement in form completion was a direct consequence of the monitoring and feedback; causality in this respect would require a randomised controlled trial of implementation—again, something that could be rendered relatively inexpensive when nested in a fully digitised infrastructure with automated randomisation and outcome measurement.

This project shows that ‘real time’ monitoring and feedback at service level may be an effective and low-cost approach to enhance the safety of prescribing of high risk drugs. This is important when comparing with traditional audit methods where evaluations may only be carried out every few years and require time-intensive manual case note review. The relatively simple NLP techniques used here have provided increased depth to the health record which could be tailored to provide additional benefits in other areas. This could improve quality in patient care and lead to significant cost savings in terms of time saved by staff carrying out laborious data collection for audits.
