# Supplementary material for: ‘Real time’ monitoring of antipsychotic prescribing in patients with dementia: a study using the Clinical Record Interactive Search (CRIS) platform to enhance safer prescribing
Source: BMJ Open Qual. 2020 Mar 29;9(1):e000778. doi: 10.1136/bmjoq-2019-000778 (PMC7170541; doi:10.1136/bmjoq-2019-000778)
Supplement: Supplementary data [file bmjoq-2019-000778supp001.pdf]

Appendix 1

Mental Health of Older Adults Initiation of antipsychotic monitoring form

Basic Details

Location*	- Please Select -		
Dementia Subtype*	- Please Select -	Severity*	- Please Select -
Other Diagnosis	- Please Select -	Any history of stroke or TIA?*	○ Yes ○ No
Is the patient currently detained under the Mental Health Act*			○ Yes ○ No

Target Symptoms

Treatment of known psychotic illness (eg schizophrenia/bipolar)*	○ Yes ○ No	Treatment of BPSD*	○ Yes ○ No

Symptoms secondary to dementia

☐ Psychosis-Delusions	☐ Psychosis-Hallucinations	☐ Fear-Anxiety	☐ Depression / Low Mood
☐ Disturbed Sleep	☐ Agitation	☐ Distress	☐ Verbal Aggression
☐ Physical Aggression	☐ Disinhibited Behaviour	☐ Resisting Help with ADL	☐ Wandering/Restlessness
☐ Vocalisation	☐ Other		

Pre - Treatment Assessment

Have the following potential medical causes of BPSD *been considered*?

Depression	☐ Yes	Anxiety	☐ Yes
Pain	☐ Yes	Side Effects of other meds	☐ Yes
Physical Illness (eg constipation, UTI, chest infection, heart failure etc..)	☐ Yes	Other Causes*	○ Yes ○ No

Other Causes Details:

Please comment briefly

Which non-pharmalogical interventions were tried before anti-psychotic?

Review of social and personal activities?	☐ Yes
Changes in staff approach? e.g. distraction, behavioural approach	☐ Yes
Changes to environment? e.g. lighting, TV, availability of quiet areas, orientation aid?	☐ Yes
Watchful Waiting / Monitoring?	☐ Yes
Other?	☐ Yes

Comments

Risk / Benefit analysis carried out (taking into account severity of BPSD, risk of side effects and stroke etc..) :

- Analysis performed*	○ Yes ○ No ○ Not applicable
**Discussion with:**	
- Relative*	○ Yes ○ No ○ Not applicable
- Professional carer*	○ Yes ○ No ○ Not applicable
- Patient*	○ Yes ○ No ○ Not applicable

Please comment briefly

Baseline Physical Monitoring

Within the last 3 months the patient had the following tests:

Blood Pressure*	◯ Yes ◯ No	*diastolic*		*systolic*		Date (tests done)	
Pulse Rate*	◯ Yes ◯ No	*bpm*				Date (tests done)	
Weight*	◯ Yes ◯ No	*kg*				Date (tests done)	
ECG*	◯ Yes ◯ No	*notes*				Date (tests done)	
Fasting blood glucose OR HbA1c*	◯ Yes ◯ No	*notes*				Date (tests done)	
Lipid Profile*	◯ Yes ◯ No	*notes*				Date (tests done)	
FBC*	◯ Yes ◯ No	*notes*				Date (tests done)	
UandEs*	◯ Yes ◯ No	*notes*				Date (tests done)	
LFTs*	◯ Yes ◯ No	*notes*				Date (tests done)	
Prolactin*	◯ Yes ◯ No	*notes*				Date (tests done)	

Abnormal Results and Actions

Start date of Drug		Antipsychotic Drug Name	
Total daily dose (mg) - Regular		Total daily dose (mg)- PRN	
Prescriber Initiating Drug	- Please Select -		

Other Medications

Appendix 2

Mental Health of Older Adults Review of antipsychotic monitoring form

Basic Details

Location*	- Please Select -		
Dementia Subtype*	- Please Select -	Severity*	- Please Select -
Other Diagnosis	- Please Select -	Any history of stroke or TIA?*	- Please Select -
Is the patient currently detained under the Mental Health Act*		○ Yes ○ No	

Target Symptoms

Treatment of known psychotic illness (eg schizophrenia/bipolar)*	○ Yes ○ No	Treatment of BPSD*	○ Yes ○ No
Symptoms secondary to dementia			

☐ Psychosis-Delusions	☐ Psychosis-Hallucinations	☐ Fear-Anxiety	☐ Depression / Low Mood
☐ Disturbed Sleep	☐ Agitation	☐ Distress	☐ Verbal Aggression
☐ Physical Aggression	☐ Disinhibited Behaviour	☐ Resisting Help with ADL	☐ Wandering/Restlessness
☐ Vocalisation	☐ Other		

Core Information

Start date of Drug		Antipsychotic Drug Name	
Total daily dose (mg)- Regular		Total daily dose (mg)- PRN	
Who initiated the most recently prescribed antipsychotic medication?		Who is currently prescribing this antipsychotic medication	- Please Select -

If the Core Information above is unclear or out of date. Please update in 'Decision Regarding Continued Treatment' section to be found further below

Discussion and Review

Risk / Benefit analysis carried out (taking into account severity of BPSD, risk of side effects and stroke etc..) :

- Analysis performed*	○ Yes ○ No ○ Not applicable
**Discussion with:**	
- Relative*	○ Yes ○ No ○ Not applicable
- Professional carer*	○ Yes ○ No ○ Not applicable
- Patient*	○ Yes ○ No ○ Not applicable
Please comment briefly	

Response to Target Symptoms

Response*	○ No change ○ Worse ○ Better ○ Not certain

Side Effects

Sedation*	○ Yes ○ No	Comments	
Falls*	○ Yes ○ No	Comments	
Impaired Mobility*	○ Yes ○ No	Comments	
Chest Infection*	○ Yes ○ No	Comments	
Anticholinergic*	○ Yes ○ No	Comments	
Rigidity*	○ Yes ○ No	Comments	
Tremor*	○ Yes ○ No	Comments	
TIA or CVA*	○ Yes ○ No	Comments	
Low Blood Pressure*	○ Yes ○ No	Comments	
Other*	○ Yes ○ No	Comments	
None*	○ Yes ○ No	Comments	

Physical health monitoring

Within the last year the patient had the following tests:

Blood Pressure*	○ Yes ○ No	*diastolic*		*systolic*	Date (tests done)	
Pulse Rate*	○ Yes ○ No	*bpm*			Date (tests done)	
Weight*	○ Yes ○ No	*kg*			Date (tests done)	
ECG*	○ Yes ○ No	*notes*			Date (tests done)	
Fasting blood glucose OR HbA1c*	○ Yes ○ No	*notes*			Date (tests done)	
Lipid Profile*	○ Yes ○ No	*notes*			Date (tests done)	
FBC*	○ Yes ○ No	*notes*			Date (tests done)	
UandEs*	○ Yes ○ No	*notes*			Date (tests done)	
LFTs*	○ Yes ○ No	*notes*			Date (tests done)	
Prolactin*	○ Yes ○ No	*notes*			Date (tests done)	

Abnormal Results and Actions

Decision Regarding Continued Treatment

Decision*	○ No change ○ Stop antipsychotic ○ Increase dose ○ Reduce dose ○ Change antipsychotic		
Decision By*		Next Review Due	
Send Review Summary to GP:	☐ Yes		
